# Optic Nerve Head Optical Coherence Tomography Angiography Findings after Coronavirus Disease

**DOI:** 10.18502/jovr.v16i4.9749

**Published:** 2021-10-25

**Authors:** Mojtaba Abrishami, Ramin Daneshvar, Zahra Emamverdian, Nasser Shoeibi, Shima Sedighi, Talieh Saeidi Rezvani, Neda Saeedian, Saeid Eslami

**Affiliations:** ^1^Eye Research Center, Mashhad University of Medical Sciences, Mashhad, Iran; ^2^Department of Emergency Medicine, University of Florida College of Medicine, Jacksonville, Jacksonville, FL, USA; ^3^Department of Education and Psychology, Ferdowsi University of Mashhad, Mashhad, Iran; ^4^Department of Internal Medicine, Faculty of Medicine, Mashhad University of Medical Sciences, Mashhad, Iran; ^5^Department of Medical Informatics, Faculty of Medicine, Mashhad University of Medical Sciences, Mashhad, Iran; ^6^Department of Medical Informatics, Amsterdam Public Health, Amsterdam UMC, University of Amsterdam, Amsterdam, The Netherlands; ^7^Pharmaceutical Research Center, School of Pharmacy, Mashhad University of Medical Sciences, Mashhad, Iran

**Keywords:** Coronavirus Disease 2019 (COVID-19), Optic Nerve Head, Optical Coherence Tomography Angiography (OCTA), Radial Peripapillary Capillary Network, Severe Acute Respiratory Syndrome Corona Virus 2 (SARS-CoV-2)

## Abstract

**Purpose:**

To quantify the microvasculature density of the optic nerve head (ONH) using optical coherence tomography angiography (OCTA) analysis in patients recovered from Coronavirus Disease 2019 (COVID-19).

**Methods:**

In a comparative cross-sectional, observational study, patients recovered from COVID-19 whose initial diagnosis was confirmed by a rRT-PCR of a nasopharyngeal sample were included in this study. OCTA of ONH was performed in included patients and normal controls. Vascular density (VD) of the all vessels (AV) and small vessels (SV) inside the disc and radial peripapillary capillary (RPC) network density were measured in COVID-19 recovered patients and compared with similar parameters in an age-matched group of normal controls.

**Results:**

Twenty-five COVID-19 patients and twenty-two age-matched normal controls were enrolled in the study and one eye per participant was evaluated. The mean whole image SV VD in the COVID-19 group (49.31 ± 1.93) was not statistically significantly different from that in the control group (49.94 ± . 2.22; *P* = 0.308). A decrease in RPC VD was found in all AV and SV VD measured, which became statistically significant in whole peripapillary SV VD, peripapillary inferior nasal SV VD, peripapillary inferior temporal SV VD, peripapillary superior nasal SV VD, and grid-based AV VD inferior sector (*P* < 0.05). Inside disc SV VD in the COVID-19 group (49.43 ± 4.96) was higher than in the control group (45.46 ± 6.22) which was statistically significant (*P* = 0.021).

**Conclusion:**

Unremarkable decrease was found in ONH microvasculature in patients who had recovered from COVID-19. These patients may be at risk of ONH vascular complications. Increase in inner disc SV VD may be an indicator of ONH hyperemia and edema.

##  INTRODUCTION

Severe acute respiratory syndrome coronavirus 2 (SARS-CoV-2) is a new member of the Coronaviridae family of viruses, which can cause serious life-threatening respiratory illness, severe pneumonia,^[1]^ and even multiorgan failure.^[2,3]^ Various clinical presentations and fatal consequences of the associated disease, Coronavirus Disease 2019 (COVID-19), have been reported, but scarce reports regarding ocular manifestations are available.^[4,5]^

Angiotensin-converting enzyme (ACE)-2 is considered as the main receptor for SARS-CoV-2 infection.^[6]^ ACE2 as a member of the renin-angiotensin-aldosterone system (RAAS) is present in different cell types including type II alveolar cells in the lung, arterial and venous endothelial cells, enterocytes of the small intestine, and smooth muscle cells of arterial vasculature of most organs.^[7]^ Its homologous enzyme is ACE, which is the main effector in the RAAS and ACE2 counterbalances and regulates its activity by reducing the amount of angiotensin-II and increasing Ang (1-7).^[8]^ ACE and ACE2 have been presented in the choroid and different cell types of the retina including retinal vascular endothelial cell, photoreceptor cells, Müller cells, and ganglion cells.^[9]^ Moreover, their expression in neurons and glial cells in the central nervous system (CNS) have been reported.^[10]^ Hence, it seems reasonable to expect ocular, and specifically optic nerve head (ONH) and CNS manifestations of the SARS-CoV-2 infection.

The majority of reports on the ocular involvement in COVID-19 describe ocular surface manifestations including conjunctival congestion, chemosis, and conjunctivitis.^[11]^ Reports of the retinal findings are numerable. Marinho and colleagues described various retinal manifestations of COVID-19 like cotton wool spots and hemorrhages.^[12]^ In our previous report, we found decrease in macular vessel density in superficial and deep capillary peluxuses of the retina, using optical coherence tomography angiography (OCTA) in otherwise healthy relatively young patients.^[13]^ However, no similar report on ONH microvascular findings was avaiable in patients infected with SARS-CoV-2.

As the choroid, retina and nervous tissues could be targets of infection, because of binding of the virus to ACE2 receptor, and previous reports on ocular posterior segment lesions in COVID-19 individuals include nonspecific findings, like retinal hemorrhages or cotton wool spots, we aimed to evaluate the ONH microvasculature in relatively young and otherwise healthy patients recovered from COVID-19 and compare it with a normal group.

##  METHODS

### Study Participants

A cross-sectional study was conducted at the Imam Reza General Hospital, Mashhad, Iran. Nurses and physicians working at Mashhad University of Medical Sciences (MUMS) with a definite diagnosis of COVID-19, confirmed by a positive test result of a nasopharyngeal swab sample real-time, reverse transcription-polymerase chain reaction, who recovered from the systemic symptoms at least two weeks prior to the enrolment, were included.

Detailed ocular and systemic histories were obtained from each participant and those with a history of autoimmune disease, migraine, diabetes mellitus, current pregnancy and breastfeeding, or any history of intraocular surgery were excluded. Additional exclusion criteria included absolute spherical refractive error > 5 diopters and cylindrical refractive error > 2 diopters, best-corrected visual acuity < 20/20, and evidence of glaucoma or clinically apparent retinal disease. Those with ocular media opacity, like cataract or corneal haziness, preventing high-quality imaging or reduced OCTA scan quality (i.e., quality scan index < 7/10) were also excluded from the analysis.

The age-matched control groups were healthy nurses and physicians employed by the MUMS who were imaged on the same OCTA machine at the Imam Reza Hospital in 2019; this group was recruited as part of an ongoing, longitudinal cohort study, PERSIAN Organizational Cohort study in MUMS.

Complete medical systemic history regarding the patients' COVID-19 symptoms, hospitalization, and disease course were recorded. Oxygen saturation at the time of examination was measured by a portable pulse oximeter.

### Image Acquisition and Analysis

OCTA scans were performed with the AngioVue (RTVue XR Avanti, Optovue, Fremont, CA, USA; Software version 2018.0.0.14), an OCTA machine with A-scan-rate of 70,000 scans/sec. Each B scan line is repeated to evaluate the image decorrelation. Optic disc cubes, AngioDisc 4.5 × 4.5 mm HD scan (400 lines × 400 A-scans) protocols, were scanned in the horizontal and vertical orthogonal directions. All measurements were primarily acquired using the automated default segmentation with the preset settings for the radial peripapillary capillary (RPC) network. The 3D Projection Artifact Removal by OCT 3D volume set were utilized.

All images were centered on the optic disc and displayed a scan quality index of at least 7/10. Images with undesirable quality or image artifact were discarded and reacquired. All images in the study, mostly segmentation accuracy, were carefully reviewed by two authors (MA, RD) to ensure sufficient quality and resolution and any images with artifact significant enough to interfere with vessel density analysis were also excluded. For all subjects, cases or controls, only the data of the eye with better image quality was used for analysis.

To evaluate the RPC layer, a slab between the outer limit of the retinal nerve fiber layer (RNFL) and the internal limiting membrane were used. All images were checked for segmentation errors and manually adjusted before testing the vessel density. All vessels (AV), including both large and small vessels (SV), and SV vascular density (VD) were evaluated separately in the RPC layer. The whole image, inside disc area, in peripapillary whole, peripapillary superior and inferior hemifields, and eight segments SV RPC VD were reported. For the evaluation of AV VD, including both large vessels and SV, the whole image was divided into nine (three by three) grid-based sections, and AV VD in all sections was reported separately. Moreover, the whole image, inside the disc area, in peripapillary whole, peripapillary superior and inferior hemifields AV were reported.

### Statistical Analysis 

The normal distribution of variables was examined using the Shapiro–Wilk test and normality plots and homogeneity of variances were ascertained by Levene's test. Based on data distribution and type, either the independent-samples *t*-test, paired *t*-tests, or Mann–Whitney U test were used for comparisons. Chi-square test and Fisher's exact test were used for categorical variables. The level of statistical significance was set at 0.05. All statistical analyses were performed using the SPSS program for Windows, version 20 (IBM SPSS Statistics, IBM Corporation, Chicago, IL, USA).

### Ethical Considerations

The study protocol adhered to the tenets of the Declaration of Helsinki. All participants provided written informed consent before enrollment and the ethical aspects of the study were approved by the Regional Committee on Medical Ethics at Mashhad University of Medical Sciences, Mashhad, Iran (IR.MUMS.REC.1399.104).

##  RESULTS

Twenty-five recovered COVID-19 patients (10 females, 40%) with a mean age of 41.5 ± 10.5 years and 22 healthy normal controls (10 females, 45.4%) with a mean age of 36.7 ± 7.3 years were enrolled in the study. Age (*P =* 0.086) and gender (*P =* 0.706) were not significantly different between the two groups. ONH parameters like cup–disc ratio and rim area were not different between the groups [Table 1]. None of the patients or controls had other systemic comorbidity except recent COVID-19 in last six months.

**Table 1:** Comparison of optic nerve head parameters and age of COVID-19 patients eyes versus normal eyes


	**Normal eyes (** ***n*** ** = 22) Mean ± SD (Range) **	**COVID-19 patients eyes (** ***n*** ** = 25) Mean ± SD (Range)**	***P*** **-value (Compare to normal)**
Age (yr)	41.5 ± 10.5 (24.00–53.00)	36.7 ± 7.3 (25.00–61.00)	0.086
Cup/Disc Area Ratio	0.14 ± 0.09 (0.00–0.33)	0.12 ± 0.10 (0.00–0.35)	0.415
Cup/Disc Vertical Ratio	0.37 ± 0.18 (0.00–0.60)	0.30 ± 0.21 (0.00–0.61)	0.283
Cup/Disc Horizontal Ratio	0.32 ± 0.16 (0.00–0.55)	0.27 ± 0.19 (0.00–0.59)	0.356
Rim Area	1.67 ± 0.27 (1.18–2.29)	1.58 ± 0.32 (1.09–2.40)	0.313
Disc Area	1.97 ± 0.31 (1.47–2.74)	1.81 ± 0.30 (1.22–2.40)	0.076
Cup Volume	0.09 ± 0.18 (0.00–0.87)	0.06 ± 0.08 (0.00–0.34)	0.469
Peripapillary RNFL	108 ± 10.77 (101.00–129.00)	112.04 ± 7.69 (83.00–133.00)	0.151
RNFL, retinal nerve fiber layer; COVID-19, Coronavirus Disease 2019; SD, Standard Deviation			

**Table 2:** Comparison of small vessels (SV) vessel density (VD) of COVID-19 patients eyes versus normal eyes


	**COVID-19 patients eyes Mean ± SD (Range)**	**Normal eyes Mean ± SD (Range)**	***P*** **-value**
Whole Image – SV VD	49.31 ± 1.93 (44.90–53.40)	49.94 ± 2.22 (44.50–53.20)	0.308
Inside Disc – SV VD	S (37.70–56.40)	45.46 ± 6.22 (24.80–53.00)	0.021
Whole PeriPapillary – SV VD	51.76 ± 1.92 (47.00–55.20)	53.14 ± 2.31 (46.00–56.70)	0.032
PeriPapillary Superior Hemi – SV VD	52.01 ± 2.16 (46.90–55.70)	53.12 ± 2.90 (44.40–57.10)	0.148
PeriPapillary Inferior Hemi – SV VD	51.79 ± 3.31 (44.90–62.00)	53.16 ± 2.13 (47.70–57.90)	0.095
PeriPapillary Nasal Superior – SV VD	49.37 ± 2.96 (42.40–53.70)	49.99 ± 3.84 ( 37.90–56.10)	0.547
PeriPapillary Nasal Inferior – SV VD	48.20 ± 4.63 (39.00–57.90)	49.52 ± 4.71 (38.00–64.50)	0.337
PeriPapillary Inferior Nasal – SV VD	48.54 ± 2.94 (42.30–52.90)	53.21 ± 4.12 (45.40–59.50)	< 0.001
PeriPapillary Inferior Temporal – SV VD	56.93 ± 3.14 (46.60–61.50)	59.78 ± 3.25 (53.60–65.20)	0.004
PeriPapillary Temporal Inferior – SV VD	52.15 ± 3.57 (47.30–60.40)	53.69 ± 3 (45.40–58.20)	0.632
PeriPapillary Temporal Superior – SV VD	55.86 ± 4.02 (44.20–61.30)	55.96 ± 4.50 (44.50–63.80)	0.889
PeriPapillary Superior Temporal – SV VD	55.24 ± 2.76 (50.50–60.20)	57.0 ± 3.92 (49.50–63.30)	0.087
PeriPapillary Superior Nasal – SV VD	49.03 ± 3.39 43.60–55.50)	51.14 ± 3.72 (41.20–58.70)	0.049
SV, small vessels; VD, vessel density; SD, Standard Deviation; COVID-19, Coronavirus Disease 2019			

**Table 3:** Comparison of all vessels (AV), including both small and large vessels, vessel density (VD) of COVID-19 patients eyes versus normal eyes


	**COVID-19 patients eyes Mean ± SD (Range)**	**Normal eyes Mean ± SD (Range)**	***P*** **-value **
Whole Image – AV VD	56.08 ± 1.89 (52.50–60.20)	56.44 ± 2.29 (51.40–59.10)	0.566
Inside Disc – AV VD	56.05 ± 4.14 (49.50–63.90)	57.25 ± 5.46 (40.40–63.80)	0.057
Whole Peripapillary – AV VD	58.31 ± 1.65 (55.00–61.80)	59.33 ± 2.34 (52.90–62.60)	0.110
Peripapillary Superior Half – AV VD	58.75 ± 1.64 (56.10–61.50)	59.50 ± 2.63 (52.00–63.70)	0.257
Peripapillary Inferior Half – AV VD	57.90 ± 2.08 (53.20–62.60)	59.11 ± 2.30 (53.90–63.60)	0.066
Grid Based AV VD Supero temporal	57.63 ± 4.21 (43.30–63.10)	59.14 ± 3.02 (52.60–64.00)	0.161
Grid Based AV VD Temporal	56.46 ± 3.08 (46.60–62.00)	57.01 ± 3.37 (50.40–61.50)	0.565
Grid Based AV VD Infero temporal	58.49 ± 4.47 (42.60–64.40)	58.67 ± 3.43 (49.20–64.10)	0.879
Grid Based AV VD Superior	56.85 ± 2.77 (52.50–62.10)	58.68 ± 3.36 (50.80–63.40)	0.051
Grid Based AV VD Central	58.10 ± 4.56 (50.00–64.60)	60.35 ± 3.23 (49.00–64.40)	0.061
Grid Based AV VD Inferior	60.68 ± 2.14 (55.30–64.50)	62.56 ± 3.15 (54.10–66.90)	0.023
Grid Based AV VD Supero nasal	51.71 ± 4.19 (43.90–58.20)	52.09 ± 3.36 (43.60–59.90)	0.739
Grid Based AV VD Nasal	52.30 ± 5.48 (43.80–67.90)	52.52 ± 3.50 (44.60–62.40)	0.869
Grid Based AV VD Inferonasal	50.29 ± 3.91 (44.40–59.80)	50.35 ± 3.84 (41.60–58.10)	0.903
AV, all vessels; VD, vessel density; SD, Standard Deviation; COVID-19, Coronavirus Disease 2019			

**Table 4:** Comparison of small vessels (SV) vessel density (VD) of hospitalized versus non-hospitalized/outpatients treated COVID-19 patients


	**Outpatients (** ***n*** ** = 16) Mean ± SD (Range)**	**Hospitalized ** ***(n*** ** = 9) Mean ± SD (Range)**	***P*** **-value**
Whole Image – SV VD	46.77 ± 4.74 (29.70–53.60)	46.45 ± 4.08 (40.30–54.00)	0.860
Inside Disc – SV VD	47.64 ± 4.89 (31.70–55.20)	47.05 ± 4.86 (38.50–55.60)	0.764
Whole PeriPapillary – SV VD	48.76 ± 4.26 (34.70–56.10)	47.10 ± 4.48 (41.70–53.20)	0.344
PeriPapillary Superior Hemi – SV VD	49 ± 4.45 (34.30–55.70)	47.48 ± 4.79 (41.50–54.20)	0.412
PeriPapillary Inferior Hemi – SV VD	47.96 ± 4.90 (33.20–56.90)	46.38 ± 4.49 (41.00–54.10)	0.415
PeriPapillary Nasal Superior – SV VD	48.90 ± 4.64 (34.40–55.90)	47.64 ± 5.11 (42.60–55.90)	0.512
PeriPapillary Nasal Inferior – SV VD	49.70 ± 3.60 (43.40–55.30)	49.85 ± 3.03 (45.70–55.70)	0.910
PeriPapillary Inferior Nasal – SV VD	49.59 ± 3.65 (42.80–54.90)	49.71 ± 2.86 (46.20–54.90)	0.930
PeriPapillary Inferior Temporal – SV VD	49.79 ± 3.69 (43.90–55.60)	50 ± 3.30 (45.20–56.40)	0.884
PeriPapillary Temporal Inferior – SV VD	32.07 ± 5.11 (18.10–40.20)	34 ± 9.32 (14.10–45.40)	0.463
PeriPapillary Temporal Superior – SV VD	52.16 ± 3.61 (45.80–58.50)	52.02 ± 3.54 (46.60–58.00)	0.919
PeriPapillary Superior Temporal – SV VD	52.16 ± 3.64 (46.30–58.20)	51.82 ± 3.38 (46.70–57.50)	0.809
PeriPapillary Superior Nasal – SV VD	52.18 ± 3.74 (45.20–58.80)	52.21 ± 3.75 (46.50–58.50)	0.987
SV, small vessels; VD, vessel density; SD, Standard Deviation; COVID-19, Coronavirus Disease 2019			

**Table 5:** Comparison of all vessels (AV), including both small and large vessels, vessel density (VD) of hospitalized versus non-hospitalized/outpatients treated COVID-19 patients


	**Non-hospitalized (** ***n*** ** = 16) Mean ± SD (Range) **	**Hospitalized (** ***n*** ** = 9) Mean ± SD (Range)**	***P*** **-value**
Whole Image – AV VD	48.58 ± 4.83 (33.40–56.30)	47.92 ± 4.28 (41.90–55.30)	0.725
Inside Disc – AV VD	49.54 ± 4.86 (31.90–56.40)	48.12 ± 3.95 (41.80–54.90)	0.444
Whole Peripapillary – AV VD	48.51 ± 4.24 (35.10–56.50)	46.68 ± 4.35 (41.60–53.00)	0.292
Peripapillary Superior Half – AV VD	45.28 ± 4.34 (30.80–53.40)	43.71 ± 4.04 (38.60–49.70)	0.362
Peripapillary Inferior Half – AV VD	52.93 ± 4.21 (40.40–59.20)	50.60 ± 4.93 (43.90–57.60)	0.197
Grid Based AV VD Superotemporal	52.41 ± 3.54 (45.30–57.80)	52.68 ± 3.02 (48.80–56.80)	0.843
Grid Based AV VD Temporal	51.53 ± 4.19 (43.90–58.20)	51.16 ± 4.06 (45.30–58.60)	0.824
Grid Based AV VD Inferotemporal	52.93 ± 3.81 (46.50–59.80)	52.22 ± 3.08 (46.70–57.40)	0.622
Grid Based AV VD Superior	51.79 ± 3.91 (44.40–58.30)	51.97 ± 4.43 (45.60–59.20)	0.911
Grid Based AV VD Central	49.87 ± 6.10 (39.50–62.90)	49.52 ± 3.44 (43.00–54.00)	0.874
Grid Based AV VD Inferior	49.88 ± 6.07 (40.30–63.50)	49.07 ± 3.21 (43.90–53.70)	0.712
Grid Based AV VD Superonasal	49.77 ± 6.24 (38.60–62.20)	49.95 ± 3.81 (42.00–54.30)	0.937
Grid Based AV VD Nasal	53.35 ± 5.56 (42.10–63.60)	53.38 ± 3.78 (46.50–58.00)	0.986
Grid Based AV VD Inferonasal	48.56 ± 6.64 (39.30–62.90)	48.66 ± 3.73 (43.30–55.10)	0.967
AV, all vessels; VD, vessel density; SD, Standard Deviation; COVID-19, Coronavirus Disease 2019			

All patients were symptom-free for at least two weeks, and the mean recovery time interval between becoming symptom free and OCTA image acquisition was 20.3 ± 2.9 days (median: 18 days; authors range: 13–29 days). Past medical history was otherwise unremarkable for almost all of the patients and controls. None of the included subjects, COVID-19 cases or controls, endorsed a history of diabetes mellitus. Two COVID-19 patients had a history of grade 1 hypertension which was well controlled with medications or diet. Nine patients (36%) had a history of hospitalization for COVID-19. O ${}_{2}$ saturation was in the normal range (94–99%) in these patients and was not different from non-hospitalized patients (*P =* 0.513). The mean scan quality was 8.28 ± 0.73 in the COVID cases and 8.50 ± 0.67 in the normal controls (*P =* 0.293). Right eye OCTA was analyzed for 11 patients and left eye for 14 patients.

**Figure 1:**
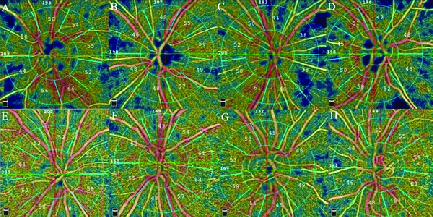
En-face optical coherence tomography angiograms (OCTA) segmented at the level of the radial peripapillary capillary (RPC) network from four recovered COVID-19 patients (A–D) versus four age-matched normal controls (E–H). Note the remarkable flow deficits present in the en-face angiograms from the COVID cases.

The 4.5 × 4.5 mm mean whole image SV VD in the COVID-19 group (49.31 ± 1.93) was not statistically different from that in the normal control group (49.94 ± 2.22) (*P =* 0.308) although it seems to be lower in the COVID-19 recovered patients [Table 2]. The 4.5 × 4.5 mm mean whole image AV VD in the COVID-19 cohort (56.08 ± 1.89) was also not statistically significantly different with that in the controls (56.44 ± 2.29) (*P* = 0.566); however, there was a tendency for lower values in the former group [Table 3; Figure 1].

Of note, whole peripapillary SV VD (51.76 ± 1.92 vs 53.14 ± 2.31; *P* = 0.032), peripapillary inferior nasal SV VD (48.54 ± 2.94 vs 53.21 ± 4.12; *P* < 0.001), peripapillary inferior temporal SV VD (56.93 ± 3.14 vs 59.78 ± 3.25; *P =* 0.004), and grid-based AV VD in inferior sector (60.68 ± 2.14 vs 62.56 ± 3.15; *P* = 0.023) were significantly lower in COVID-19 patients, compared with normal controls. In addition, in all other parameters, except inside disc SV VD, COVID-19 patients had lower VD in AV and SV VD, but the differences were not statistically significant. Inside disc SV VD in COVID-19 patients (49.43 ± 4.96) was higher than the inside disc SV VD in the normal control group (45.46 ± 6.22), which was statistically significant (*P* = 0.021).

Moreover, subgroup analysis between hospitalized and outpatient treated patients revealed no statistically significant difference between two groups although unremarkable lower values were observed in hospitalized patients versus outpatients [Tables 4 and 5].

##  DISCUSSION

In the present study, we used OCTA to determine the VD in the ONH and the RPC layer of optic disc in a relatively young, mild involvement, without other comorbidity and minority hospitalized, cohort of recovered COVID-19 patients and compare it with an age-matched normal control group. Except inside disc SV VD which was higher in the COVID-19 patients, in all RPC measured SV and AV VD recovered COVID-19 patients showed lower values, which became statistically significant in whole peripapillary SV VD, peripapillary inferior nasal SV VD, peripapillary inferior temporal SV VD, and grid-based AV VD Inferior sector. In other segments of peripapillary SV VD and grid-based sectors of AV VD, vessel densities tend to be lower in the COVID group but this did not reach statistical significance. In a subgroup analysis, in hospitalized patients we found in all RPC measured SV and AV VD unremarkable lower values versus outpatient treated COVID-19 patients which were not statistically significant.

In an autopsy study, Casagrande and associates detected SARS-CoV-2 viral particles in the retina of patients who had deceased due to COVID-19.^[14]^ Presence of ACE2 receptors in CNS and various layers of the retina and choroid have been reported;^[9,10]^ hence, pathologic changes in the ocular tissues, and especially in ONH and retina, may be expected.

In a recent case series using Zeiss Cirrus 5000 HD OCT Angioplex, RPC plexus perfusion density was lower in 80 COVID-19 recovered patients compared to 30 controls. Moreover, patients treated with lopinavir, ritonavir, or antiplatelet therapy during admission had lower RPC flow index and RPC perfusion density.^[15]^ It indicates probable ONH involvement in COVID-19. Moreover, Guillain-Barré syndrome and Miller–Fisher-like syndrome have also been reported in association with SARS-CoV-2 infection.^[16,17]^ In recent studies, SARS-CoV-2 potential for neuroinvasion has been suggested.^[18]^ It seems that in-line with the previous reports of retinitis and optic neuritis induced by coronaviruses in animal models,^[19]^ and the proposed neuroinvasion hypothesis, SARS-CoV-2 can cause neuro-inflammation and neuro-infection in humans too. Besides the neurologic involvements, as retinal ganglion cells, Müller cells, and blood vessels are potential targets of the virus, the ONH and retinal findings in COVID-19 are well anticipated. Vascular changes can be either primary or secondary to alternations in the hemodynamic demands of the inflamed and damaged tissues.

Macular OCTA analysis also found similar results. In another study by our group, using Optovue AngioVue Imaging System, macular VD was decreased in COVID-19 patients compared with normal controls.^[13]^ In another similar study in Turkey with a same machine, parafoveal VD in the superior and nasal quadrants of the superficial capillary plexus and in all quadrants of the deep capillary plexus were significantly lower in COVID-19 patients.^[20]^ Moreover, in a case–control study in patients stratified to mild, moderate, and severe COVID-19 disease activity, and normal controls, patients with moderate and severe disease had decreased macular VD as compared with control subjects or even those who were asymptomatic or paucisymptomatic.^[21]^ It has been proposed that SARS-CoV-2 infection, as a systemic multi-organ infection, may affect retinal vasculature beside other organs and marks retinal microvasculature.^[22]^

OCTA analysis is an invaluable tool in the evaluation of vascular changes in the retina and ONH for diagnosis, staging, and monitoring in glaucoma.^[23,24]^ In addition, in several neurologic conditions, including preclinical Alzheimer's disease, OCTA was found to be helpful.^[25]^ In other neurodegenerative diseases and even mild cognitive disorders, OCTA has been proposed as an additional biomarker for the early diagnosis and disease activity monitoring.^[26,27]^ In this study, we found that ONH microvasculature was somewhat decreased compared to the age-matched controls. In all segments, VD was numerically decreased in these comparative analyses, although the differences were statistically significant in some segments.

An intriguing finding in our study was the higher SV inside disc VD, which was statistically higher than normal controls. Previously, it has been reported that ONH, nerve fiber layer thickness, is increased in COVID 19 recovered patients.^[28,29]^ In a case report, acute bilateral optic neuritis has been reported in a 44-year-old Hispanic male patient with no past medical history. Multimodal ocular, orbit, and CNS imaging and paraclinic evaluations were performed to determine the possibility of demyelinating or other autoimmune disease and these evaluations were negative as well as other laboratory assessment. Therefore, it has been concluded that his infection with COVID-19 virus has triggered his immune system to present ONH findings.^[30]^ Besides our findings, increase in SV VD inside the disc area, which is in contrary to RPC VD findings, may be associated with ONH edema and hyperemia, indicating potential optic nerve involvement in COVID-19 patients, in line with our previous findings of nerve fiber layer thickening in COVID-19 patients.

Essentially, the retina and optic nerve are considered to be intraorbital extensions of the CNS, so evaluation of the retinal, and more specifically ONH changes, may help us to identify the CNS disease as well. Indeed, ONH and pRNFL changes have been already reported in patients with CNS pathologies like Alzheimer, multiple sclerosis, and diabetic polyneuropathy.^[31,32,33]^ In a recent review, neuro–ophthalmic manifestations of COVID-19 was divided to afferent and efferent manifestations.^[34]^ The examples of the afferent complications include papilledema, optic neuritis, papillophlebitis, and even vision loss due to stroke, while the efferent complications include ocular myasthenia gravis, cranial neuropathies, Miller Fisher syndrome, and Adie's pupils.^[34]^ These presentations magnifies the importance of ONH evaluations in COVID-19 patients, as these presentations beyond ophthalmic importance which may affect visual function of patients, should be considered as CNS involvement which may be more disabling or even fatal.

Our control group was recruited from healthy personnel of MUMS, as part of a longitudinal cohort study, PERSIAN Organizational Cohort study in MUMS.^[35]^ The controls were evaluated and OCTA images were acquired to build a local normal OCTA normative database and cohort analysis. The individuals in the control group were examined when no COVID-19 cases were formally reported in Iran in 2019. Therefore, there is no chance for presence of any symptom-free patient among our control group.

This study has several limitations. First, OCTA images were acquired during the recovery of the disease and not during the acute phase when the systemic condition was active. Second, longitudinal analysis of the patients was not performed. Third, we had a limited sample size, which can explain the lack of statistical significance in some comparisons. A larger-scale study with OCTA performed during the symptomatic phase of the disease, followed by repeat imaging at fixed intervals, would provide valuable information regarding both the short and long-term effects of COVID-19 on the ONH vascular system.

To the best of our knowledge, it's the first report of ONH OCTA findings in patients with a history of COVID-19. The clinical relevance of our finding is unclear, as the patients were all asymptomatic with 20/20 vision at the time of this analysis. This can indicate the acute inflammatory phase of the ocular involvement and the associated vasodilation. It is noteworthy that the systemic and ocular disease phases can be unparalleled and occur at different times. Nevertheless, our findings in a relatively young group of COVID-19 confirmed patients who had no other comorbidity, and the comparison with an age-matched normal control group, similarly imaged, is novel and may highlight the importance of continued vigilance for the detection of nervous tissue and ocular complications associated to COVID-19 as the pandemic evolves.

In conclusion, our study demonstrated ONH VD alterations in patients with a history of COVID-19 and without other comorbidities, including a decrease in AV and SV RPC VD. These findings beside previous microvascular retinal findings in these patients indicate potential retinal and ONH ischemic changes, which may be a manifestation of CNS potential vascular involvement. Moreover, increase in inside disc SV VD may be an indicator of ONH hyperemia and edema.

##  Financial Support and Sponsorship

The authors would like to acknowledge the financial support of the Vice-Chancellor of Research of Mashhad University of Medical Sciences for this research project (code: 990069). The funding organization had no role in the design or conduct of this study.

##  Conflicts of Interest

The authors declare no potential conflicts of interest for the research, authorship, and/or publication of this article.
